# Diagnostic approach to elevated dd-cfDNA with reassuring EMB in heart transplantation

**DOI:** 10.3389/frtra.2025.1623514

**Published:** 2025-08-14

**Authors:** Rupinder K. Bahniwal, Aditya Mehta, Jamie L. W. Kennedy

**Affiliations:** Department of Cardiovascular Disease, Inova Schar Heart and Vascular, Falls Church, VA, United States

**Keywords:** biomarkers, transplantation, rejection, monitoring, donor-specific antibodies, cell-free DNA, gene expression

## Abstract

Despite significant advances in organ preservation, surgical techniques, and immunosuppressive regimens, rejection continues to pose a major challenge in the care of heart transplant patients. Endomyocardial biopsy (EMB) remains the gold standard test for surveillance and diagnosis of rejection, but is limited by its invasiveness, interobserver variability, procedural risk, and cost thus prompting the widespread use of non-invasive biomarkers such as donor-derived cell-free DNA (dd-cfDNA). Due to its high negative predictive value, dd-cfDNA is often routinely used for surveillance of asymptomatic patients. However, it is a non-specific marker of allograft injury and elevated levels in the presence of a reassuring EMB creates a diagnostic dilemma. This review explores the pathophysiological basis and clinical utility of dd-cfDNA in monitoring of heart transplant recipients with particular focus on evaluation and management of discordant findings.

## Introduction

Heart transplantation is the definitive treatment for patients with heart disease refractory to conventional medical, surgical, and device therapies. Despite advances in organ preservation, surgical techniques, immunosuppressive therapies, and longitudinal care, the survival rates post heart transplant (HT) have only marginally increased in the last decade (1). Patient survival is often limited by the competing problems of rejection and infection, and complications of immunosuppression such as kidney disease and malignancy. The precise calibration of immunosuppressive therapies to mitigate these risks remains a complex challenge in the management of transplant recipients. Clinically, patients with acute rejection (AR) can present with a wide spectrum of findings, ranging from asymptomatic rejection detected on routine surveillance testing to cardiogenic shock or sudden death (2). Since its introduction in the 1970s, the EMB has been the cornerstone of AR detection, allowing for histopathological evaluation of myocardial tissue and grading of rejection based on the criteria established by the International Society for Heart and Lung Transplantation (3–5). However, it is an invasive procedure associated with inherent risks such as cardiac arrhythmias (0.25%) (6), perforation (0.1%–0.5%) (7–9), damage to the tricuspid valve apparatus (0.27%–6.3%) (10, 11), and in some cases the risk of sedation. There is also significant variability in its histological and immunopathological interpretation (12, 13). To minimize the risk of a false negative test, multiple EMB specimens (usually 3) are obtained. Despite this, false negatives do exist either through sampling error, sample preservation artifacts, confusion with quilty lesions, or biopsy negative rejection (BNR) (14). BNR is an uncommon clinical diagnosis defined by the presence of allograft dysfunction without evidence of AR on histopathology, either cellular (ACR) or antibody-mediated (AMR) (15).

To address these limitations, the field of HT has witnessed tremendous growth in the development of non-invasive strategies promising earlier, safer, and more consistent detection of rejection in the last decade (16). Most commonly used in clinical practice is donor-derived cell-free DNA (dd-cfDNA), which assesses the fragments of DNA released into the recipient's bloodstream from apoptotic and necrotic donor cells (17). Elevated levels of dd-cfDNA have been shown to correlate with episodes of ACR and AMR (18–20). Due to its high negative predictive value (>98%), its widespread use in recent years has led to a significant reduction in the frequency of routine surveillance EMBs (18, 20). Nevertheless, in either asymptomatic patients biopsied following an abnormal dd-cfDNA result, or for cause biopsies in response to patient symptoms or other evidence of graft dysfunction, discordant results namely abnormal dd-cfDNA without evidence of AR on biopsy represent a diagnostic dilemma. In broader context, is the dd-cfDNA a false positive, is the biopsy a false negative, or is there another process causing allograft injury?

This review aims to explore the evolving role of dd-cfDNA in the monitoring of heart transplant recipients, emphasizing its strengths, limitations, and clinical implications when used in conjunction with EMB. We also propose a multimodal diagnostic framework to enhance the understanding of this clinical dilemma and optimize care for patients with HT in this complex and rapidly evolving field.

### Donor-derived cell-free DNA: mechanism, diagnostic role, and limitations

Cell-free DNA is released into the bloodstream with cell death due to normal cell turnover or disease processes (21). Normally, healthy individuals have small amounts of cfDNA, most of which is released by hematopoietic cells with a small amount released from the heart (22). In organ transplant recipients, it was hypothesized that acute rejection leads to cell death in the allograft resulting in elevated levels of dd-cfDNA in the blood of transplant recipients (17). The clinical use of earlier techniques such as shot-gun sequencing and targeted quantification using polymerase chain reaction were impractical due to cost, complexity, requirement of previous genotyping of both donor and recipient, and sex-mismatch between the donor and recipient (23). These limitations were overcome with the advent of targeted amplification. Allosure (CareDx, Inc., Brisbane, CA) is a targeted amplification sequencing assay which has been analytically and clinically validated to quantify the percentage of dd-cfDNA in the transplant recipients’ blood (18). This technique was first utilized in the D-OAR study to target highly polymorphic single nucleotide polymorphisms (SNPs) to distinguish dd-cfDNA from recipient-derived cfDNA (rd-cfDNA) (24). The Allosure assay currently includes a panel of 405 SNPs. Another commercially available assay is the Prospera test (Natera, Inc., Austin, TX), validated for clinical use in the DEDUCE trial in 2021, and includes more than 13,000 SNPs (20). Table 1 summarizes the commercially available and research grade dd-cfDNA assays.

**Table 1:** Summary of commercially available and research grade dd-cfDNA assays.

Assay Name & Developer	Type	Technology	SNPs	Key Trials/Studies	Cut-off Value	Sensitivity & Specificity	NPV	Key Findings
AlloSure Heart (Care Dx)	Commercial	Targeted NGS (SNP-based)	405 SNPs	D-OAR (Khush et al., 2019)	0.2%	44% and 80%	97.1%	dd-cfDNA ≥ 0.2% associated with acute rejection; high NPV; can reduce biopsy frequency
Prospera Heart (Natera)	Commercial	Targeted NGS (SNP-based)	> 13,000 SNPs	DEFINE-HT (Shah et al., 2025) DEDUCE (Kim et al., 2021)	0.15% - 0.23% 0.15%	Not published 79% and 77%	Not published 97.3%	dd-cfDNA predicts graft dysfunction better than biopsy; strong correlation with adverse outcomes AUC 0.86 for AR in > 800 samples; high NPV; robust multicenter validation
Shotgun Whole Genome Sequencing (WGS)	Research-grade	Shotgun NGS	NA	GRAfT (Agbor-Enoh et al., 2021)	0.25%	80% and 76%	99%	dd-cfDNA < 0.25% could avoid 81% of biopsies with < 1% missed rejection; detects injury before histology
QX200 AutoDG Droplet Digital PCR (Bio-Rad Laboratories)	Research-grade	Droplet Digital PCR	35 SNPs	Böhmer et al., 2023	7.5 copies/mL	92% and 43%	92%	Absolute quantification; sensitivity of 92% with dd-cfDNA cutoffs (0.1% donor fraction, 7.5 copies/mL)

ACR, acute cellular rejection; AMR, antibody-mediated rejection; AUC, area under the curve; cfDNA, cell-free DNA; dd-cfDNA, donor-derived cell-free DNA; ddPCR, droplet digital polymerase chain reaction; DEDUCE, Donor-derived cfDNA Evaluation for the Detection of Cardiac Allograft Rejection; DEFINE-HT, Diagnostic Evaluation of Donor-derived cfDNA In Heart Transplantation; D-OAR, Donor-derived Cell-Free DNA-Outcomes AlloMap Registry; GRAfT, Genomic Research Alliance for Transplantation; NGS, next-generation sequencing; NPV, negative predictive value; SNP, single nucleotide polymorphism.

**Figure 1:**
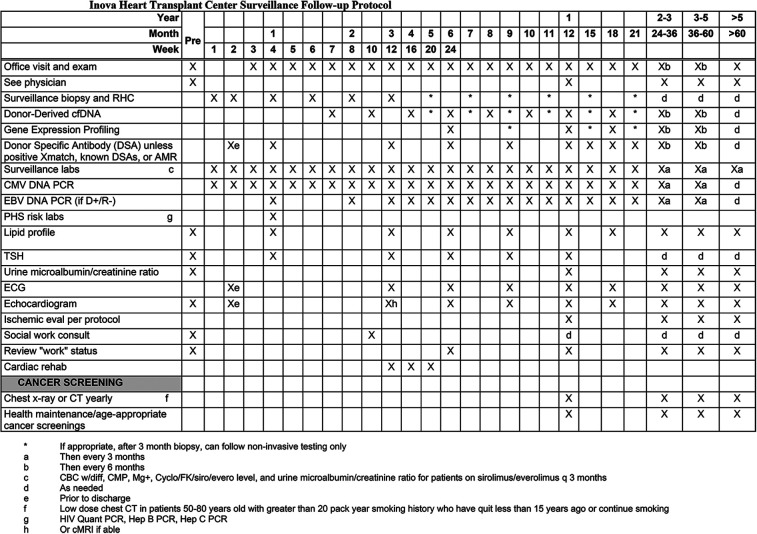
Routine surveillance protocol for patients after heart transplantation at Inova Schar Heart and Vascular (ISHV) Hospital. The figure shows the routine surveillance protocol for patients after heart transplantation. RHC with EMB at weeks 1, 2, 4, 6, 8, and 12. Dd-cfDNA at weeks 7 and 10, then monthly for months 4 through 12. Surveillance echocardiogram at weeks 2, then at months 3, 6, 9, and 12. DSAs at weeks 2 and 4, followed by at months 3, 6, 9, and 12. AMR, antibody mediated rejection; cfDNA, cell-free DNA; CMV, cytomegalovirus; EBV, Epstein Barr virus; PCR, polymerase chain reaction; PHS, public health service; TSH, thyroid stimulating hormone; ECG, electrocardiogram.

The advantages of dd-cfDNA testing include its noninvasive nature, potential for early detection, and opportunity for longitudinal monitoring. Elevated percentage of dd-cfDNA levels have been correlated with ACR and AMR, as well as other forms of allograft injury (25). Prior studies have demonstrated that an elevated dd-cfDNA fraction can precede biopsy-proven rejection by up to 3 months, highlighting its important role in screening allografts in routine surveillance, especially in the first year post-transplant (17, 19, 26). Apart from earlier detection, researchers from Genomic Research Alliance for Transplantation (GRAfT) consortium demonstrated that dd-cfDNA from patients with AMR were rich in guanosine-cytosine bases, quantitatively 5–11 times higher, and often detected sub-clinical AMR (19). By utilizing dd-cfDNA, up to 80% of the routine surveillance biopsies could be eliminated, highlighting the high NPV (>98%) of the test (19). Elevated dd-cfDNA fraction also offers prognostic information as higher levels have been linked to unfavorable outcomes including allograft dysfunction and death at one year (13). However, it is important to note that dd-cfDNA is non-specific marker of graft injury, therefore it is used as a screening tool for AR while EMB is the test of choice in cases of suspected rejection (27). Figure 1 shows the surveillance protocol for HT recipients at our center.

### Non-rejection causes of elevated donor-derived cell-free DNA

The lack of histological evidence of rejection on EMB has typically been reassuring to clinicians, but the interpretation of elevated dd-cfDNA fraction in the setting of a “negative” EMB is unclear. Plausible explanations for the discordance include subclinical rejection, non-rejection allograft injury, false positive dd-cfDNA, and false negative EMB. In instances where percentages of dd-cfDNA are elevated despite a negative biopsy, it is imperative to systematically consider these possibilities. This should include an initial assessment of technical reasons for dd-cfDNA fraction to be falsely elevated, such as improper handling of the sample, artifactual increase due to recent cardiac procedure such as biopsy or pacemaker implantation, or even after vigorous exercise (19, 28, 29). The absolute quantity of cfDNA levels is subject to large variations and have been reported in leukocytosis, systemic lupus erythematosus, myocardial infarction, and psychiatric disorders (30–33). Significant reduction in total rd-cfDNA can increase the dd-cfDNA fraction without cardiac injury. Pre-transplant considerations such as an autonomic storm in brain-dead donors or donor coronary artery disease can also lead to baseline cellular injury in the donor heart contributing to elevations in dd-cfDNA fraction in the recipient immediately post HT (34). Additionally, factors such as ischemic time, surgical complexity, pulmonary vascular resistance, and crossmatch status are speculated to lead to elevations in dd-cfDNA immediately post HT. To mitigate or reduce the likelihood of false positives, centers have adopted protocols where the timing of initial dd-cfDNA testing occurs 28 days following transplant (see Figure 1) (19).

If there are no such technical reasons, then a closer look at the immunosuppression trough levels and medication adherence is critical. Viral infections, most commonly cytomegalovirus (CMV), are associated with increased dd-cfDNA levels (27, 35, 36). CMV frequently causes leukopenia which may lead to reduction in rd-cfDNA and resulting increase in percentage of dd-cfDNA. Afzal et al. reported 4 clusters in their single center study of patients with elevated dd-cfDNA without biopsy evidence of AR: CMV viremia, non-CMV infections (which included SARS-CoV-2, Ebstein-Barr virus, and hepatitis B infection), females (with 40% having *de novo* DSA), and lastly patients with right ventricular dysfunction (27). Of note, ACR 1R was present in all patients with RV dysfunction, 80% of females, and none in the non-CMV infection group. It is unclear whether any patients with ACR 1R progressed to higher-grade rejection, but it will be important to study in future larger multicenter studies as currently most episodes of ACR 1R are not treated. However, in the D-OAR study by Kanwar et al., CMV infections were not associated with significantly higher percentages of dd-cfDNA as compared to those without CMV infection (36). Our group has observed transient elevations in dd-cfDNA immediately following vaccinations.

**Figure 2:**
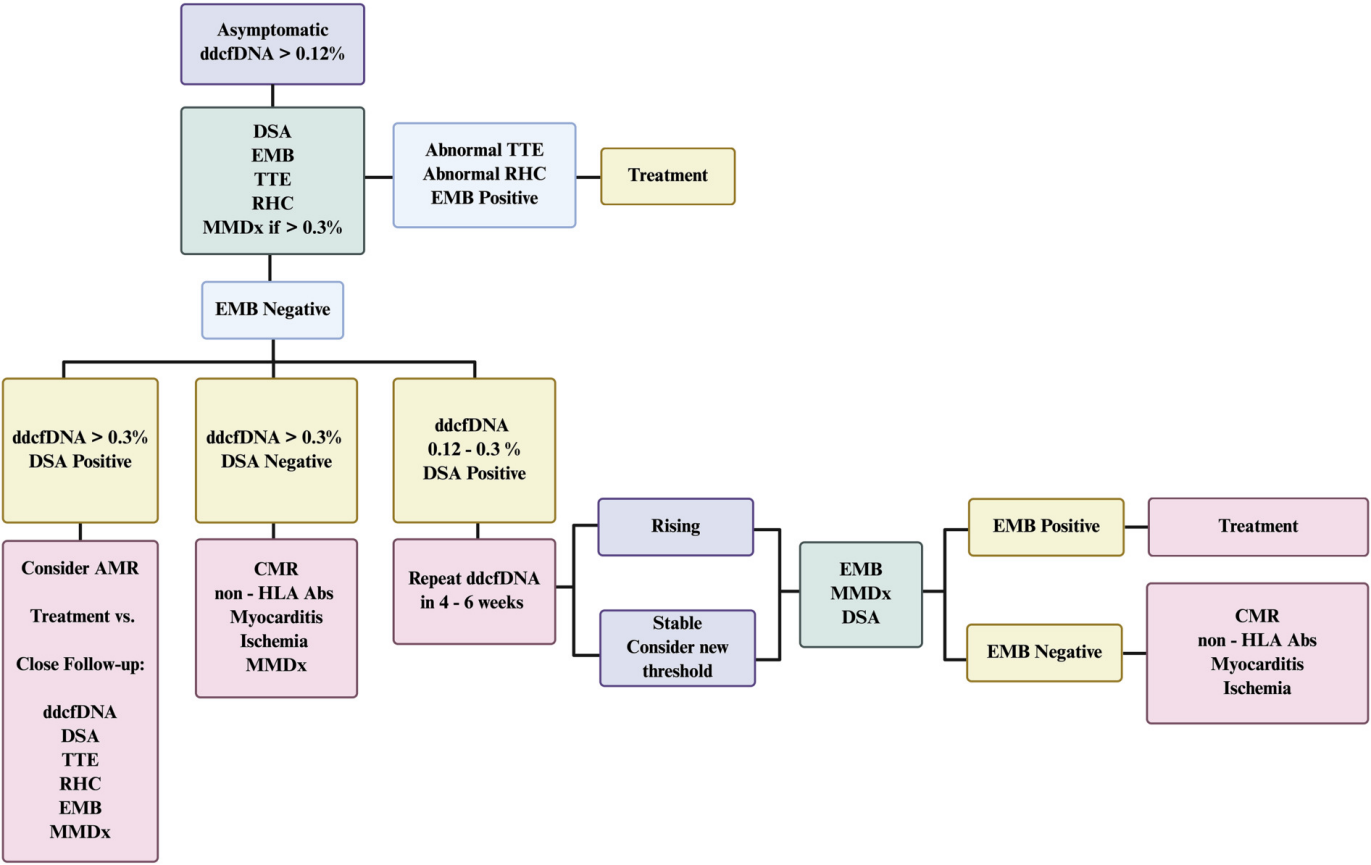
Suggested algorithm for management of asymptomatic patients with elevated dd-cfDNA without biopsy-proven rejection. The figure outlines a stepwise approach to the evaluation and management of an asymptomatic heart transplant recipient with elevated donor-derived cell-free DNA. In cases where dd-cfDNA is elevated but endomyocardial biopsy (EMB) results are negative, the algorithm incorporates a multimodal diagnostic strategy to guide further assessment and clinical decision-making. Close follow-up of possible AMR would include subsequent testing with dd-cfDNA, DSAs, TTE, RHC with EMB and MMDx. AMR, antibody-mediated rejection; CMR, cardiac magnetic resonance; dd-cfDNA, donor-derived cell-free DNA; DSA, donor-specific antibodies; EMB, endomyocardial biopsy; HLA, human leukocyte antigen; MMDx, molecular microscope diagnostic system; RHC, right heart catheterization; TTE, transthoracic echocardiogram.

Elevated dd-cfDNA fraction may reflect ischemic injury secondary to cardiac allograft vasculopathy (CAV) (34). Therefore, further ischemic evaluation with coronary angiography or noninvasive techniques should be considered. Finally, myocarditis is a possible etiology of non-rejection myocardial injury. Elevated percentage of dd-cfDNA due to toxoplasmosis has been described (37). Recurrent sarcoidosis and giant cell myocarditis following transplant are well-established, presumably they could also cause an elevated dd-cfDNA fraction. A high index of suspicion is required to make these diagnoses along with close collaboration with pathologists. To evaluate these possibilities, the incorporation of supplemental diagnostic techniques is warranted, as outlined in the central illustration (Figure 2). We, briefly discuss the role of multimodal diagnostics in evaluation of such cases when dd-cfDNA fraction is elevated in the absence of biopsy-proven rejection.

Additional biomarkers have been investigated to predict AR. Early work using troponin T and I to diagnose AR was not encouraging and recent efforts with high sensitivity assays have been shown to be more promising (38). Similarly, B type natriuretic peptide and its fragments correlated poorly with AR, though patient level trending was noted to be more informative (38). Current ISHLT guidelines state these biomarkers may have a role in a comprehensive strategy for rejection surveillance (38). The combination of dd-cfDNA fraction and gene expression profiling (GEP) has been extensively studied with incremental improvement in predicting AR on biopsy (39). A recent publication combined dd-cfDNA with a readily available biomarker NT-proBNP to improve accuracy in predicting rejection (40). Both these studies relied on EMB as the gold standard for diagnosing rejection, despite its known limitations, and neither study addressed the clinical dilemma of elevated dd-cfDNA levels in the context of reassuring biopsy findings.

The Molecular Microscope Diagnostic System (MMDx, Thermo Fisher Scientific, Waltham, MA) analyzes EMBs using microarrays to quantify mRNA transcripts associated with rejection and injury (41). It is unique in that is uses machine-based learning algorithms to compare each biopsy against a reference database of samples and follows the principle that depending on the type and severity of transplanted organ injury, specific genes are activated and produce unique mRNA patterns (42). While MMDx does not replace histology, it can provide additional information to reconcile discordant results. A contemporary study included more than 3,200 EMBs to identify 7 rejection archetypes as well as non-rejection injury and atrophy-fibrosis (43). The ongoing Trifecta-Heart study seeks to incorporate all three tools (dd-cfDNA, MMDx, and EMB histopathology) to improve accuracy in diagnosing AR (NCT04707872) (44).

Donor specific antibodies (DSAs), primarily targeting donor human leukocyte antigen molecules, are associated with AMR and poor graft survival (45). DSAs may be present at the time of transplant, or may develop following transplant termed *de novo*. The development of *de novo* DSA(s) or significant increase in the strength of known DSA(s) coupled with allograft dysfunction even in the presence of a negative EMB may warrant treatment. Moreover, antibodies against non-HLA antigens such as angiotensin II type-1 receptor and others have been linked to AMR and BNR (46, 47); however, monitoring is highly variable across centers, and standardized treatment protocols remain poorly defined.

Cardiac MRI is another promising non-invasive alternative surveillance tool for AR in HT. Besides assessing graft function and valve lesions, parametric mapping with T1 and T2 sequences can potentially enable detection of myocardial edema, fibrosis, and interstitial expansion. In a small study of 60 HT patients, T2 relaxation has been shown to be an independent predictor of AR (48). Another small randomized trial comparing EMB and CMR for AR surveillance demonstrated high diagnostic accuracy of CMR achieving 92% sensitivity and 92% NPV for AR (49). However, CMR is not able to distinguish between different types of AR. CMR can also aid in the diagnosis of myocarditis, including toxoplasmosis, giant cell, and sarcoidosis.

### Future directions

As the clinical utility of dd-cfDNA continues to evolve in HT surveillance, several avenues hold promise for advancing the field and addressing current diagnostic limitations. Transitioning from relative quantification or donor fraction to absolute quantification of dd-cfDNA, may enhance diagnostic specificity and reduce variability across diverse clinical scenarios. However, absolute level of dd-cfDNA has also been correlated to AR providing a stable, reproducible measure of donor-specific injury (18, 50–52). This approach may be particularly beneficial in patients with high baseline cfDNA due to concurrent illness or those with multi-organ transplants, where interpreting percentage of dd-cfDNA can be challenging. The primary results of the DEFINE-HT (Development of Non-Invasive Cell-Free DNA to Supplant Invasive Biopsy in Heart Transplantation) study recently reported that Prospera (Natera, Inc, Austin, TX) with donor quantity score (DQS) incorporating both absolute dd-cfDNA and donor fraction, predicted allograft dysfunction 3 times more than EMB and demonstrated strong correlation with clinical outcomes at one year (53).

The use of dd-cfDNA in multiorgan transplant recipients is not well-described. Dd-cfDNA fractions of healthy allografts vary by the transplanted organ, the heart is typically the lowest at < 0.20%, kidney < 0.5% (54), lung < 1% (55), and liver the highest ranging from 5 to 30% (56), which makes selecting a threshold for multiorgan recipients challenging. Intriguingly, dd-cfDNA can be fractionated if recipients have organs from multiple donors, permitting one to follow each organ independently (57). If the transplanted organs are from a single donor however, setting an appropriate threshold must be done on a case-by-case basis, which has been reported in the literature (58) and we have done at our center.

Integration of dd-cfDNA with other emerging diagnostics, such as MMDx, CMR, and DSA profiling, represents an important step toward a precision medicine approach in HT. By combining molecular, immunological, and imaging data, clinicians may be able to generate a more comprehensive and nuanced risk profile for each patient, enabling more timely and targeted interventions. Lastly, longitudinal studies are needed to understand the kinetics of dd-cfDNA over time, establish optimal surveillance intervals, and determine the clinical significance of transient elevations in the absence of rejection.

## Conclusion

Elevated dd-cfDNA in the context of a negative EMB poses a significant diagnostic challenge, highlighting the shift from traditional reliance on EMB towards an integrated, multimodal diagnostic approach for AR surveillance in the contemporary era. While dd-cfDNA offers the advantage of non-invasive testing with high NPV, its interpretation must consider the broader clinical context, including other potential causes of graft injury and limitations of the “gold standard” EMB. Future research should focus on refining multimodal diagnostic algorithms to improve early detection and management of AR, ultimately personalizing the diagnostic testing for individual patients based on their clinical characteristics and, thereby improving overall outcomes for patients with HT.
